# Supplementary material for: V1 superficial layers create a saliency map that feeds forward to the parietal cortex for attentional guidance
Source: bioRxiv. 2025 Apr 10:2025.04.10.648136. Preprint. [Version 1] doi: 10.1101/2025.04.10.648136 (PMC12190818; doi:10.1101/2025.04.10.648136)
Supplement: 1 [file NIHPP2025.04.10.648136V1-supplement-1.pdf]

bioRxiv preprint doi: https://doi.org/10.1101/2025.04.10.648136; this version posted April 10, 2025. The copyright holder for this preprint (which was not certified by peer review) is the author/funder, who has granted bioRxiv a license to display the preprint in perpetuity. It is made available under aCC-BY 4.0 International license.

# Supplementary figures

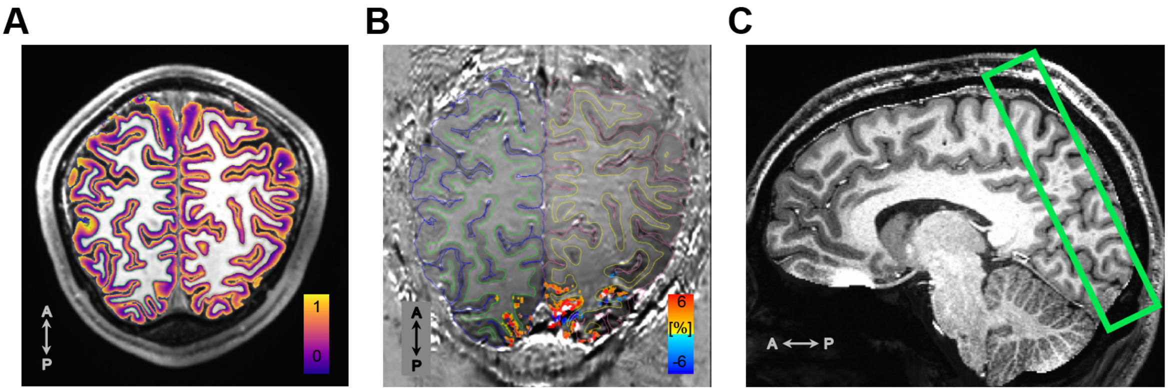


**Figure S1.** (A) Normalized cortical depth map overlayed on the T1w anatomical image in a representative participant. The equi-volume depth at 0 and 1 correspond to the WM and Pial surfaces, respectively. (B) VASO activations (90°+15°+0°, $p < 0.001$ uncorrected) overlayed on the mean VASO image. Green and yellow lines indicate the WM surface, while the blue and pink lines denote the pial surface. (C) The green box indicates the 3D slab of VASO fMRI acquisition.

bioRxiv preprint doi: https://doi.org/10.1101/2025.04.10.648136; this version posted April 10, 2025. The copyright holder for this preprint (which was not certified by peer review) is the author/funder, who has granted bioRxiv a license to display the preprint in perpetuity. It is made available under aCC-BY 4.0 International license.

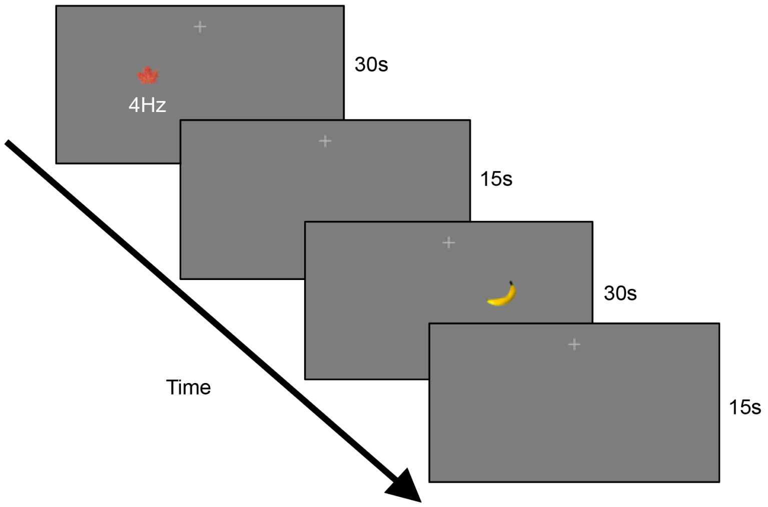


**Figure S2.** Stimulus and procedure of the localizer runs. Naturalistic stimuli were presented at 4 images per second in the lower-left or lower-right quadrants in separate stimulus blocks, interleaved with 15-second fixation periods. The size and location of localizer stimuli matched the foreground region in the main experiment.

bioRxiv preprint doi: https://doi.org/10.1101/2025.04.10.648136; this version posted April 10, 2025. The copyright holder for this preprint (which was not certified by peer review) is the author/funder, who has granted bioRxiv a license to display the preprint in perpetuity. It is made available under aCC-BY 4.0 International license.

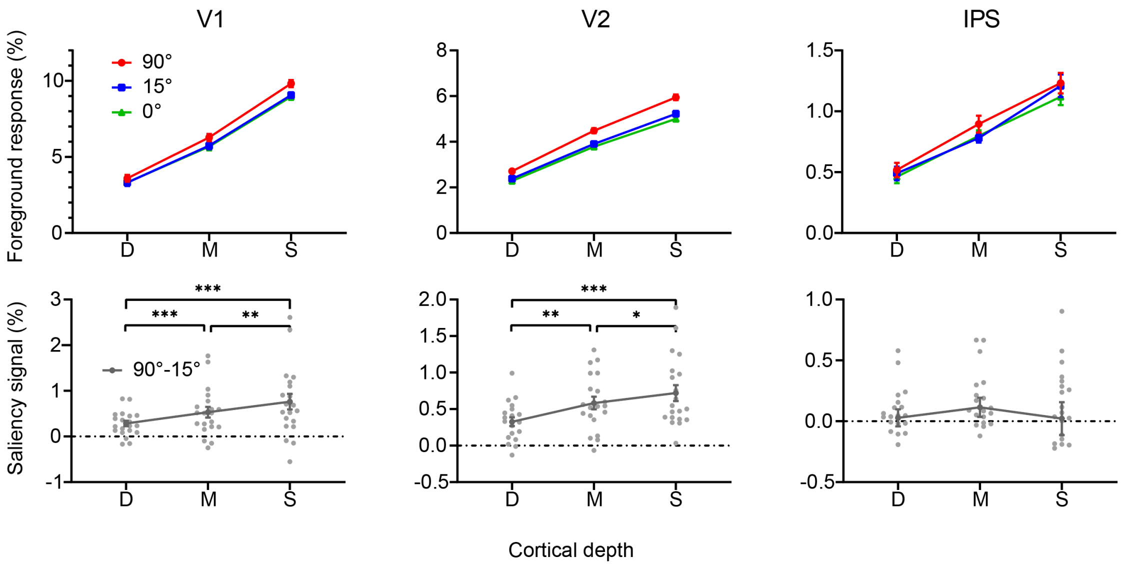


**Figure S3.** Normalized BOLD responses in the foreground ROIs of V1, V2, and IPS. Top panel: BOLD response in different depths of V1, V2, and IPS in 90°, 15°, and 0° orientation contrast conditions; Bottom panel: Calculated from top panel, the response difference between 90° and 0° foregrounds. Error bars represent the standard deviation of the mean. *, ** and *** indicate $p < 0.05$, $p < 0.01$, $p < 0.001$.

bioRxiv preprint doi: https://doi.org/10.1101/2025.04.10.648136; this version posted April 10, 2025. The copyright holder for this preprint (which was not certified by peer review) is the author/funder, who has granted bioRxiv a license to display the preprint in perpetuity. It is made available under aCC-BY 4.0 International license.

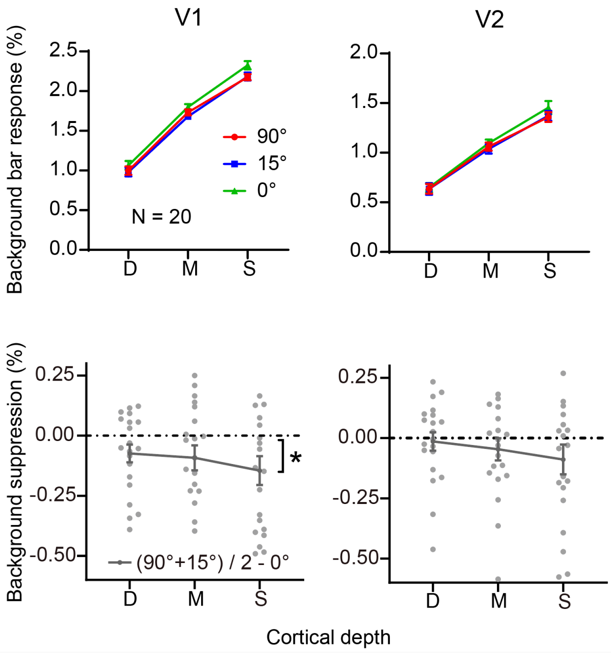


**Figure S4.** Upper: normalized CBV responses in the background ROIs of V1 and V2. A significant effect of orientation contrast (θ = 90°, 15°, and 0°) was found in V1 ($F_{2, 38}$ = 3.700, p = 0.034) and a similar trend in V2, suggesting weaker background activity in the 90° and 15° conditions compared to the 0° or the uniform texture condition. No significant difference was found between the two θ conditions ($F_{1, 19}$ = 0.365, p = 0.553, $BF_{10} = 5.601\times10^{-11}$). Lower: The suppression effect was calculated as the response difference between the mean of 90° and 15° conditions and the 0° condition ((90°+15°)/2-0°). A significant suppression effect was found only in the superficial depth of V1 (t19 = -2.877, p = 0.023, Holm corrected across cortical depths). These results suggest a suppression effect of background activity in the superficial layers of V1, independent with the orientation contrast between the foreground and the background bars. Each gray dot represents one participant. Error bars indicate SEM. * indicates p < 0.05. D, M, S indicate deep, middle, and superficial depth, respectively.

bioRxiv preprint doi: https://doi.org/10.1101/2025.04.10.648136; this version posted April 10, 2025. The copyright holder for this preprint (which was not certified by peer review) is the author/funder, who has granted bioRxiv a license to display the preprint in perpetuity. It is made available under aCC-BY 4.0 International license.

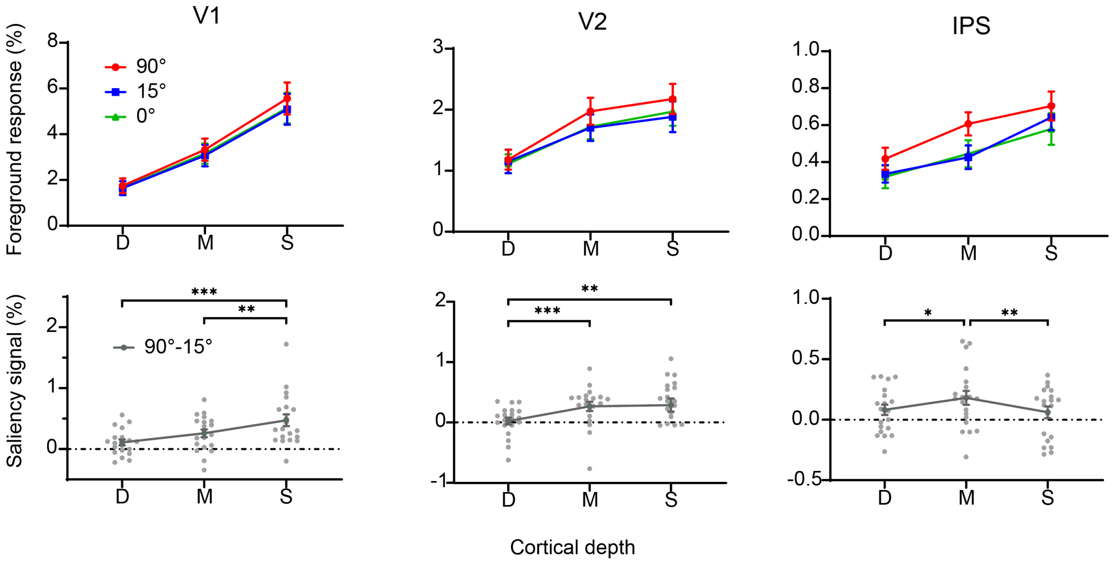


**Figure S5.** Unnormalized (or original) CBV responses in the foreground ROIs. Conventions are identical as in Figure 3A.

bioRxiv preprint doi: https://doi.org/10.1101/2025.04.10.648136; this version posted April 10, 2025. The copyright holder for this preprint (which was not certified by peer review) is the author/funder, who has granted bioRxiv a license to display the preprint in perpetuity. It is made available under aCC-BY 4.0 International license.

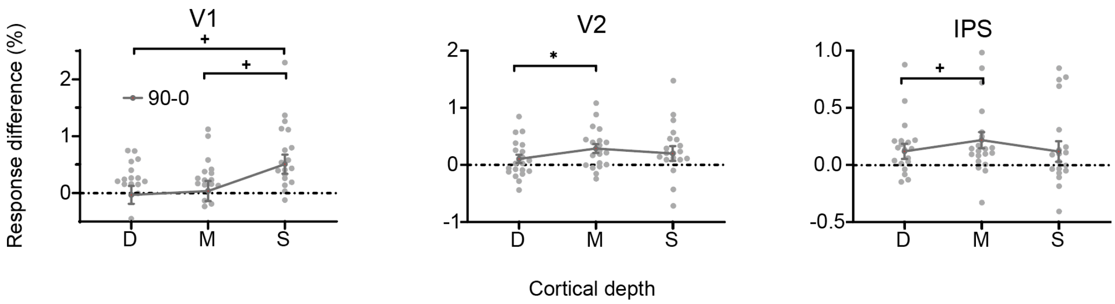


877

878 **Figure S6.** Normalized CBV response difference in the foreground ROIs between 90° and 0°

879 orientation contrast conditions. Similar laminar profile with Figure 3A bottom panel. Error bars

880 represent the standard error of the mean. * $p < 0.05$, + $p < 0.1$.

bioRxiv preprint doi: https://doi.org/10.1101/2025.04.10.648136; this version posted April 10, 2025. The copyright holder for this preprint (which was not certified by peer review) is the author/funder, who has granted bioRxiv a license to display the preprint in perpetuity. It is made available under aCC-BY 4.0 International license.

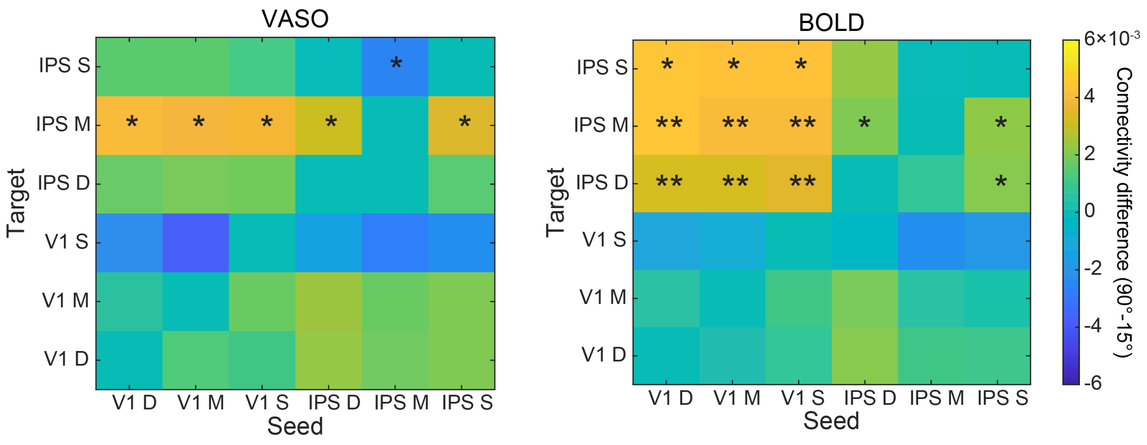


**Figure S7.** The gPPI connectivity matrix across three cortical depths in V1 and IPS. Columns and rows correspond to the seed and target ROIs, respectively. The color scale indicates the beta difference of interaction terms (beta(90°)-beta(15°)). * $p < 0.05$, ** $p < 0.01$, uncorrected.

bioRxiv preprint doi: https://doi.org/10.1101/2025.04.10.648136; this version posted April 10, 2025. The copyright holder for this preprint (which was not certified by peer review) is the author/funder, who has granted bioRxiv a license to display the preprint in perpetuity. It is made available under aCC-BY 4.0 International license.

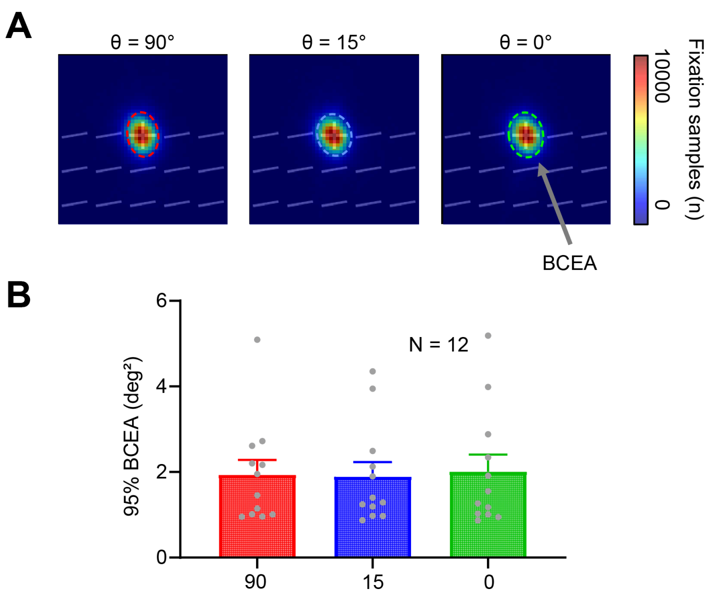


**Figure S8.** (A) The group-averaged heat maps of fixation distribution in the entire session. (B) The bivariate contour ellipse area (BCEA) of fixation distribution showed no significant difference across θ conditions ($F_{2,22} = 0.291$, $p = 0.750$, $BF_{10} = 0.227$). Error bars represent the standard deviation of the means.

bioRxiv preprint doi: https://doi.org/10.1101/2025.04.10.648136; this version posted April 10, 2025. The copyright holder for this preprint (which was not certified by peer review) is the author/funder, who has granted bioRxiv a license to display the preprint in perpetuity. It is made available under aCC-BY 4.0 International license.

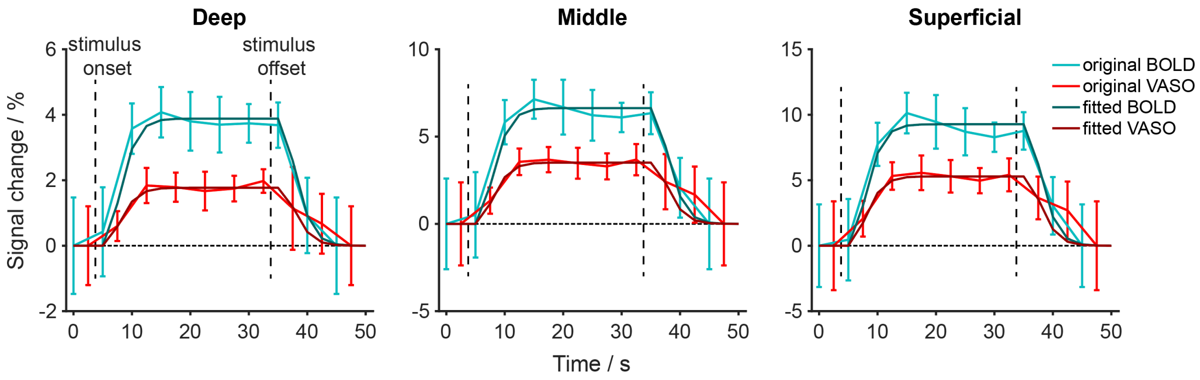


**Figure S9.** VASO and BOLD response timecourses in deep, middle, and superficial ROIs of the foreground in V1, and the fitted response with GLM using a canonical HRF (BLOCK4 in AFNI). Error bars represent SEM across participants.
